# Inflammation and the risk of atrial high-rate episodes (AHREs) in patients with cardiac implantable electronic devices

**DOI:** 10.1007/s00392-018-1244-0

**Published:** 2018-04-17

**Authors:** Daniele Pastori, Kazuo Miyazawa, Yanguang Li, Farhan Shahid, Hussein Hado, Gregory Y. H. Lip

**Affiliations:** 1 Institute of Cardiovascular Sciences, University of Birmingham, Birmingham, UK; 2 Department of Internal Medicine and Medical Specialties, I Clinica Medica, Atherothrombosis Center, Sapienza University of Rome, Rome, Italy; 3 Department of Cardiology, Chinese PLA Medical School, Chinese PLA General Hospital, Beijing, China; 4 Cardiology Department, City Hospital, Birmingham, B18 7QH UK; 5 Aalborg Thrombosis Research Unit, Department of Clinical Medicine, Aalborg University, Aalborg, Denmark

**Keywords:** AHREs, Implantable device, Pacemaker, Atrial fibrillation, Inflammation

## Abstract

**Introduction:**

Atrial high-rate episodes (AHREs) are associated with an increased risk of developing atrial fibrillation and thromboembolism. The characteristics of ‘real world’ patients developing AHREs are poorly known.

**Methods:**

We included 496 consecutive patients with cardiac implantable electronic devices (CIEDs). Primary endpoint was occurrence of AHREs, defined as > 175 bpm and lasting > 5 min, in a median follow-up of 16.5 (IQR 3.9–38.6) months (1082.4 patient-years). We also tested the predictive value of clinical risk scores for AHREs.

**Results:**

Mean age was 68.8 ± 14.0 years, and 35.5% were women; AHREs were recorded in 173 patients [34.7%, 16.0%/year, 95% confidence interval (CI) 13.7–18.6]. Multivariable Cox regression analysis showed that age [hazard ratio (HR) 1.020, 95% CI 1.004–1.035, *p* = 0.011], prior AF (HR 3.521, 95% CI 2.831–5.206, *p* < 0.001), white cell count (HR 1.039, 95% CI 1.007–1.072, *p* = 0.016) and high C reactive protein (CRP; HR 1.039, 95% CI 1.021–2.056, *p* = 0.038) were independently associated with AHREs. ROC curve analysis showed that the APPLE score (*C* statistic 0.53, 95% CI 0.48–0.59; *p* = 0.296) ALARMEc score (*C* statistic 0.51, 95% CI 0.44–0.57; *p* = 0.810) were non-significantly associated with AHRE. Similar results were obtained for CHADS_2_ and CHA_2_DS_2_VASc score

**Conclusion:**

AHREs are common in CIEDs patients, with age, prior AF, inflammatory markers (high CRP, white cell count) being factors associated with AHREs onset. Clinical risk scores showed limited value for AHREs prediction in this cohort.

**Electronic supplementary material:**

The online version of this article (10.1007/s00392-018-1244-0) contains supplementary material, which is available to authorized users.

## Introduction

Previous studies showed that atrial high-rate episodes (AHREs) are associated with an increased risk of new-onset atrial fibrillation (AF) [1], thromboembolism [2–4] and cardiovascular mortality [5]. Subclinical ischaemic brain lesions have also been described in patients with AHREs [6]. However, the thromboembolic risk of AHRE may be lower when compared to patients with AF, and a significant proportion of ischemic strokes recorded in patients with AHREs do not show a significant temporal relationship with AHREs occurrence [7].

Although implicated to play role in cryptogenic stroke, the clinical significance of AHREs is not well understood and the management of these patients is not evidence-based. Furthermore, clinical characteristics and risk factors associated with the development of AHREs are poorly described.

Conventional diagnostic methods, such as resting ECG and Holter monitoring have limited value in the detection of paroxysmal AF and AHREs [8]. Conversely, current use of cardiac implanted electronic devices (CIEDs), including pacemakers and implantable defibrillators lead to an improvement in the early detection of atrial and ventricular arrhythmic episodes, especially in patients that are asymptomatic [9]. AHREs lasting > 5 min are considered as clinically relevant, and patients presenting with AHREs should be assessed for the presence of other risk factors for stroke, and regularly screened to detect overt AF to consider of antithrombotic therapy [10].

We investigated incidence and factors associated with the development of AHREs in a cohort of consecutive patients who underwent CIEDs implantation.

## Methods

We included all consecutive patients with CIEDs including DDD pacemaker, implantable cardioverter defibrillator (ICD), or cardiac resynchronization therapy (CRT) device, who attended the Cardiology Department of Sandwell and West Birmingham Hospitals NHS Trust (Sandwell General Hospital and City Hospital) in Birmingham, United Kingdom from December 2010 to August 2017. We excluded patients with single-chamber VVI devices and patients with < 3 months of follow-up. The atrial sensitivity was programmed to 0.5 mV with bipolar sensing.

At baseline, personal medical history and information on co-morbidities and concomitant medications were collected. The primary endpoint for the study was the occurrence of AHRE, defined as > 175 bpm and lasting > 5 min. Baseline clinical characteristics of patients with and without AHREs were compared. This study was conducted in accordance with the EU Guidance on Good Clinical Practice CPMP/ECH/135/95 and the Declaration of Helsinki.

### Clinical risk scores

We tested the predictive value of clinical risk scores in predicting the occurrence of AHREs. The various scores were calculated as follows:

(i) APPLE score: Age [> 65 years (A), persistent AF (P), impaired eGFR (< 60 ml/min/1.73 m^2^) (P), LA diameter ≥ 43 mm (L), and EF < 50% (E). Each variable scored 1 point with the score ranging from 0 to 5 points;
(ii) ALARMEc score: AF type (A), Left Atrial size [normalized left atrial area (NLA) ≥ 10.25], Renal insufficiency (eGRF < 68 ml/min), Metabolic syndrome and cardiomyopathy (c) with each variable scoring 1 point, and the score values ranging from 0 to 5 points. The ALARMEc score was calculated in a subgroup of 250 patients with complete information on metabolic syndrome. For APPLE and ALARMEc scores left atrial enlargement was calculated as a normalized left atrial volume/body surface area > 28 ml/m^2^.
(iii) CHADS_2_ score: congestive heart failure, hypertension, age ≥ 75, diabetes mellitus, prior stroke or TIA. Each variable was assigned 1 point, prior stroke or TIA was scored 2 points, ranging from 0 to 6 points;
(iv) CHA_2_DS_2_-VASc score: congestive heart failure, hypertension, age ≥ 75, diabetes mellitus, prior stroke or transient ischemic attack, vascular disease, age 65 to 74, female; 2 points assigned to age ≥ 75 and prior stroke or TIA; 1 point to every other variable, ranging from 0 to 9 points.

### Statistical analysis

Continuous variables were expressed as mean ± standard deviation (SD) or median and interquartile range (IQR), as appropriate. Categorical variables were expressed as numbers and percentages. The Pearson *χ*^2^ test was used to compare proportions.

Cox proportional hazards analyses were used to calculate the adjusted relative hazard ratio (HR) by each clinical variable. In the multivariable Cox regression model, only variables with a *p* < 0.100 at univariate analysis were entered. Statistical significance was set at a *p* value < 0.05.

We also tested the predictive value of clinical risk scores such as APPLE, ALARMEc, CHADS_2_ and CHA_2_DS_2_VASc score. To investigate the predictive performance (expressed as c-indexes) of these scores, we used receiver operating characteristic (ROC) curves, and compared them as described by DeLong et al. [11]. All tests were two-tailed and analyses were performed using computer software packages (IBM SPSS 23.0 for Windows^®^, SPSS Inc.) and MedCalc^®^ V. 18.4.1.

## Results

We included 496 consecutive patients undergoing CIEDs in this study. Indications for CIEDs implantation included sick sinus syndrome (22.2%), heart block (34.9%), heart failure (26.3%), ventricular tachycardia/fibrillation (13.0%), other (2.8%, e.g., recurrent/malignant vasovagal syncope, symptomatic bradycardia), and unknown (0.8%, 4 patients).

Mean age was 68.8 ± 14.0 years, and 35.5% were women. Table 1 reports baseline characteristics of patients with and without AHREs. Patients with detected AHREs were older, with a higher prevalence of prior AF (and accordingly, higher use of oral anticoagulants), digoxin use, higher bilirubin and white cell count (Table 1).

**Table 1:** Baseline characteristics of patients according to AHREs occurrence

	AHRE (≥ 5 min)		*p* value
	No (*n* = 323)	Yes (*n* = 173)	
Women (%)	37.5	31.8	0.238
Age (years)	67.9 ± 14.4	70.3 ± 13.2	0.078
AF (any type) (%)	9.8	39.5	< 0.001
Paroxysmal AF (%)	6.4	26.9	
Persistent AF (%)	0.6	5.3	
Permanent AF (%)	2.6	7.6	
Hypertension (%)	66.8	65.3	0.763
Diabetes (%)	30.1	29.1	0.837
Heart Failure (%)	40.8	37.4	0.498
CAD (%)	41.6	36.8	0.333
Stroke/TIA (%)	9.2	12.8	0.219
Metabolic syndrome (*n* = 250, %)	46.6	41.2	0.438
Waist circumference (*n* = 250, cm)	104.3 ± 14.7	102.5 ± 14.8	0.335
LA enlargement (*n* = 348, %)	57.4	62.9	0.368
Ejection fraction (*n* = 358, %)	45.4 ± 18.6	48.4 ± 16.8	0.119
Haemoglobin (g/l)	132.3 ± 18.4	133.3 ± 18.3	0.573
Platelet count (× 10^9^/l)	241.8 ± 76.6	229.1 ± 63.6	0.064
White cell count (× 10^9^/l)	7.8 ± 2.4	8.7 ± 5.7	0.011
CRP (mg/l)	3.0 (2.0–10)	5.0 (2.5–11.5)	0.150
High CRP (above median) (%)	47.9	56.6	0.099
Creatinine (μmol/l)	100.4 ± 55.0	99.2 ± 42.7	0.797
eGFR classes			0.480
> 90 ml/min/1.7	18.9	16.2	
89–50 ml/min/1.7	50.9	50.3	
49–30 ml/min/1.7	25.5	30.6	
< 30 ml/min/1.7	4.7	2.9	
eGFR < 68 ml/min/1.7 (%)	47.5	50.3	0.572
eGFR < 60 ml/min/1.7 (%)	30.2	33.5	0.477
ALT (U/l)	28.2 ± 25.9	29.0 ± 32.0	0.786
Potassium (mmol/l)	4.6 ± 2.4	4.5 ± 0.5	0.460
Bilirubin (μmol/l)	11.7 ± 6.8	13.0 ± 9.9	0.048
Beta Blockers (%)	40.2	42.4	0.699
ACEI/ARB (%)	57.0	57.6	0.924
Diuretic (%)	43.4	50.0	0.181
Oral anticoagulant (%)	13.0	36.8	< 0.001
Antiplatelet (%)	49.4	40.4	0.058
Digoxin (%)	3.8	11.8	0.002
Amiodarone (%)	8.9	9.4	0.869
Statin (%)	63.0	62.4	0.922
Calcium channel blocker (%)	19.9	18.2	0.718

*ACEI* angiotensin-converting enzyme inhibitor, *ARB* angiotensin receptor blocker, *AF* atrial fibrillation, *ALT* alanine aminotransferase, *CAD* coronary artery disease (previous myocardial infarction, cardiac revascularization), *CRP* C reactive protein, *eGFR* estimated glomerular filtration rate, *LA* left atrium, *TIA* transient ischaemic attack

### Predictors of AHREs

28 patients with missing data at follow-up were excluded from this analysis. Hence, 471 CIEDs patients were included in the Cox regression model. Overall, 173 AHREs (34.7%) were recorded during a median follow-up of 16.5 (IQR 3.9–38.6) months yielding 1082.4 patient-years of observation (incidence rate 16.0%/year, 95% CI 13.7–18.6).

Based on results from univariate Cox regression analysis (Table 2), age, atrial fibrillation, stroke/TIA, white cell count, bilirubin, diuretic, antiplatelet, and digoxin were used as covariates in the multivariable model. The variable “oral anticoagulation” was excluded from the final model as > 90% of these patients were already classified as “having AF”. However, in a fully adjusted model “oral anticoagulation” was not significant, and this exclusion did not modify final results (data not shown). The multivariable Cox regression analysis showed that age [hazard ratio (HR) 1.020, 95% CI 1.004–1.035, *p* = 0.011], prior AF (HR 3.521, 95% CI 2.831–5.206, *p* < 0.001), white cell count (HR 1.039, 95% CI 1.007–1.072, *p* = 0.016) and high CRP (above median, HR 1.039, 95% CI 1.021–2.056, *p* = 0.038) were independently associated with AHREs onset (Table 2).

**Table 2:** Cox regression analysis for AHREs predictors

	Univariate				Multivariate			
	Hazard ratio	95% confidence interval		*p* value	Hazard ratio	95% confidence interval		*p* value
Age	1.023	1.011	1.035	< 0.001	1.020	1.004	1.035	0.011
Female sex	0.860	0.620	1.194	0.369				
Atrial fibrillation	4.877	3.556	6.690	< 0.001	3.521	2.831	5.206	< 0.001
Hypertension	1.101	0.795	1.523	0.563				
Diabetes	1.104	0.790	1.542	0.562				
Heart failure	0.904	0.659	1.241	0.533				
Stroke/TIA	1.507	0.959	2.369	0.075	1.458	0.902	2.355	0.124
Coronary artery disease	0.885	0.645	1.215	0.450				
Haemoglobin	0.994	0.985	1.002	0.153				
Platelet count	0.999	0.996	1.001	0.276				
White cell count	1.048	1.021	1.077	0.001	1.039	1.007	1.072	0.016
High CRP	1.547	1.108	2.159	0.010	1.449	1.021	2.056	0.038
Creatinine	1.000	0.996	1.004	0.932				
ALT	0.998	0.991	1.006	0.674				
Potassium	0.924	0.748	1.140	0.461				
Bilirubin	1.022	1.002	1.042	0.031	1.006	0.986	1.027	0.572
Beta blockers	1.016	0.744	1.386	0.922				
ACEI/ARB	1.029	0.753	1.406	0.858				
Diuretic	1.308	0.963	1.778	0.086	0.887	0.619	1.271	0.514
Oral anticoagulation	3.855	2.803	5.302	< 0.001				
Antiplatelet	0.688	0.504	0.940	0.019	0.783	0.539	1.137	0.199
Digoxin	2.217	1.388	3.541	0.001	1.357	0.791	2.330	0.268
Amiodarone	0.912	0.543	1.532	0.727				
Statin	1.019	0.741	1.402	0.906				
Calcium channel blockers	1.173	0.793	1.737	0.424				

See Table 1 for abbreviations

### Clinical risk scores for AHREs

Clinical risk scores showed only modest and statistically non-significant predictive ability for AHREs. ROC curves for each score are shown in supplementary Fig. 1. The APPLE Score was tested on 348 patients with 132 AHREs, and had a *C* statistic of 0.53 (95% CI 0.48–0.59) *p* = 0.296. The ALARMEc score was tested on 233 patients with 93 incident AHREs and a *C* statistic of 0.51 (95% CI 0.44–0.57) *p* = 0.810. In this subgroup where metabolic syndrome could be defined, the C statistics for the APPLE score was 0.56, 95% CI 0.50–0.63; *p* = 0.054. Similar non-significant results were obtained for CHADS_2_ and CHA_2_DS_2_ VASc_2_ scores (see supplementary Fig. 1).

## Discussion

The main findings of this ‘real world’ cohort study are as follows: (1) more than 30% of CIEDs recipients have device-detected AHREs, (2) presence of AF, older age, high CRP and white cell count all independently predicted incident AHREs, and (3) clinical risk scores have generally limited value for AHREs prediction.

The reported incidence of AHRE in previous studies ranges from 30 to 70% according to different definitions used [2]. Some studies defined AHRE by a rate between 170 and 225 bpm, with the duration of the episode > 20 s or > 5–6 min [12]. In our cohort, 34.6% of patients had a detected AHRE, with an incidence rate of 16.0%/year. This percentage is similar to that observed in the ASSERT study, which included 2580 patients with CIEDs and no history of AF, and demonstrated that the AHREs lasting more than 6 min were found in 34.7% of the patients over a mean follow-up of 2.5 years [1].

We found that increasing age, AF, high CRP and white blood cell count were all independently associated with AHRE occurrence. While age and the presence of supraventricular arrhythmia, such as AF, have been already described as predictors of AHRE/AF, we—as far as we are aware—are the first to describe association between increased inflammatory markers and incidence of AHREs with a direct association between peri-implantation inflammatory biomarkers (CRP and white cell count) and incidence of AHREs during follow-up.

Similarly, a previous study has shown that high post-cardiac surgery white cell count was associated with an increased risk of post-operative AF [13]. This finding reinforces the association between inflammation and the occurrence/recurrence of atrial arrhythmia and may represent a common pathogenetic pathway linking AHREs to AF [14]. Thus, upon activation, white cells produce a number of inflammatory mediators, such as cytokines and reactive oxidant species, which interact with cardiomyocytes of atrial tissue leading to electrical remodelling and fibrosis development [15]. The presence of AHREs may represent an early manifestation of this (pro-inflammatory) process.

Moreover, inflammatory mediators favour to a pro-thrombotic state [16, 17], which may account for the increased risk of thromboembolism and cardiovascular mortality also in this setting. Hence, the relationship between inflammation and cardiovascular events in patients with AHREs deserves further research.

Interestingly, we found no significant association between anti-arrhythmic drugs, such as amiodarone, beta blockers or digoxin, and AHRE incidence. This result suggests that the optimal management of patients with AHREs is still to find, and further research is needed to optimize anti-arrhythmic therapy to minimize the burden of silent arrhythmic episodes.

Several clinical risk scores have been proposed to predict new-onset atrial tachyarrhythmias [18]. However, most were done on small cohorts and had no certain external validation. In our cohort, we tested two of these scores, namely the APPLE and ALARMEc scores, and our results suggest that both have limited predictive value.

The coexistence of prior AF in up to 40% of patients developing AHRE indicates the close link between the two conditions, representing the clinical continuum of atrial tachyarrhythmias. This may support the need for stroke prevention in patients with AHREs which is currently under evaluation in randomized control trials; two ongoing trials are exploring this issue [19, 20]. Apart from CIEDs, increasingly sophisticated means of monitoring (e.g., implanted loop recorders) to detect AHREs in various clinical settings and high-risk groups, that would merit consideration of stroke prevention, have been recently investigated [21–23]. Of note, a recent consensus document from the European Heart Rhythm Association recommended that patients presenting with subclinical atrial tachyarrhythmias (i.e., AHRE > 5 min) should be considered for oral anticoagulation when ≥ 2 stroke risk factors using the CHA_2_DS_2_-VASc score are present [24].

### Limitations

The study has some limitations. This was a single-centre study performed in hospital-based setting. As the atrial amplitude during AF decreases, the incidence of AHRE may be underestimated, leading to missed AHRE or to separate a long AHRE episode into multiple brief episodes. Additionally, we did not store all electrogram of AHREs, but device diagnostic information on AHREs was reviewed by at least one experienced electrophysiologist whether they were true AHREs or other device-oversensing events, blinded to clinical outcomes.

In conclusion, AHREs are common in CIEDs patients, with age, prior AF and inflammatory indexes (high CRP, white cell count) being factors associated with AHREs onset. Clinical risk scores showed limited value for AHREs prediction in this cohort.

## Electronic supplementary material

Below is the link to the electronic supplementary material.

Supplementary material 1 (DOCX 23 KB)
