# Supplementary material for: Inflammation and the risk of atrial high-rate episodes (AHREs) in patients with cardiac implantable electronic devices
Source: Clin Res Cardiol. 2018 Apr 17;107(9):772–7. doi: 10.1007/s00392-018-1244-0 (PMC6105258; doi:10.1007/s00392-018-1244-0)
Supplement: Supplementary file 1 — Supplementary material 1 (DOCX 23 KB) [file 392_2018_1244_MOESM1_ESM.docx]

**Inflammation and the risk of atrial high-rate episodes (AHREs) in patients with cardiac implantable electronic devices.**

**Supplementary data**

**Supplementary Figure 1. ROC curve analysis for clinical risk scores towards AHREs detection. Apple score (Panel A), ALARMEc (Panel B), CHAD_2_ VASc_2_ score (Panel C), CHADS_2_ (Panel D).**

| **APPLE Score on 348 patients with 132 AHREs**  C statistics 0.53 (0.48-0.59) p=0.296. | **ALARMEc Score** **on 233 patients and 93 AHREs**  C statistics 0.51 (0.44-0.57) p=0.810 |
| --- | --- |
|  |  |
| **CHAD_2_ VASc_2_ score**  C statistics 0.51 (0.46-0.55) p=0.757 | **CHADS_2_ score**  C statistics 0.53 (0.48-0.57) p=0.293 |
|  |  |
